# Genome Structure, Evolution, and Host Shift of *Nosema*

**DOI:** 10.3390/biology13110952

**Published:** 2024-11-19

**Authors:** Xiao Xiong, Christopher J. Geden, Yongjun Tan, Ying Zhang, Dapeng Zhang, John H. Werren, Xu Wang

**Affiliations:** 1 Department of Pathobiology, College of Veterinary Medicine, Auburn University, Auburn, AL 36849, USA; xzx0019@auburn.edu (X.X.); yzz0207@auburn.edu (Y.Z.); 2 Center for Medical, Agricultural and Veterinary Entomology, USDA Agricultural Research Service, Gainesville, FL 32608, USA; chris.geden@usda.gov; 3 Department of Biology, College of Arts & Sciences, Saint Louis University, St. Louis, MO 63103, USA; yongjun.tan@slu.edu (Y.T.); dapeng.zhang@slu.edu (D.Z.); 4 Department of Biology, University of Rochester, Rochester, NY 14627, USA; jack.werren@rochester.edu; 5 Alabama Agricultural Experiment Station, Center for Advanced Science, Innovation and Commerce, Auburn, AL 36849, USA; 6 HudsonAlpha Institute for Biotechnology, Huntsville, AL 35806, USA

**Keywords:** fungal genomics, microsporidia, telomeric repeats, genome reduction, cis-regulatory motif, codon bias

## Abstract

*Nosema* is a diverse fungal genus of unicellular, obligate symbionts infecting various arthropods. We performed comparative genomic analyses of seven *Nosema* species that infect bees, wasps, moths, butterflies, and amphipods. As intracellular parasites, these species exhibit significant genome reduction, retaining only about half of the genes found in free-living yeast genomes. Notably, genes related to oxidative phosphorylation are entirely absent (*p* < 0.001), and those associated with endocytosis are significantly diminished compared to other pathways (*p* < 0.05). All seven *Nosema* genomes display significantly lower G-C content compared to their microsporidian outgroup. Species-specific 5~12 bp motifs were identified immediately upstream of start codons for coding genes in all species (*p* ≤ 1.6 × 10^−72^). Our RNA-seq data from *Nosema muscidifuracis* showed that this motif is enriched in highly expressed genes but depleted in lowly expressed ones (*p* < 0.05), suggesting it functions as a *cis*-regulatory element in gene expression. We also discovered diverse telomeric repeats within the genus. Phylogenomic analyses revealed two major *Nosema* clades and incongruency between the *Nosema* species tree and their hosts’ phylogeny, indicating potential host switch events (100% bootstrap values). This study advances the understanding of genomic architecture, gene regulation, and evolution of *Nosema*, offering valuable insights for developing strategies to control these microbial pathogens.

## Simple Summary

A group of fungal parasites called *Nosema* infects various insects, including bees, wasps, butterflies, moths, and even some crustaceans. These parasites are harmful because they can weaken or kill their hosts, posing a significant threat to beneficial insects. Our aim is to understand how *Nosema* genomes have evolved and to find potential ways to control them. These parasites were discovered to have much smaller genomes compared to free-living organisms like yeast, missing about half of the genes, especially those involved in energy production and certain cellular processes, suggesting they rely heavily on their hosts to survive. Interestingly, we found evidence that *Nosema* species have jumped between hosts in their evolutionary history, including butterflies, wasps, and bees. Additionally, we identified specific regulatory DNA sequences common in highly active genes, indicating potential roles in controlling gene expression. Understanding these genomic features can help us develop strategies to manage *Nosema* infections, which is valuable for protecting bees and other pollinators, as well as wasps that act as natural biological control agents for agricultural pests. This research provides important insights into protecting insect populations that are crucial for ecosystems and agriculture, thereby supporting food production and maintaining healthy ecosystems.

## 1. Introduction

Microsporidia are a diverse group of unicellular parasites that infect a wide range of animals [1,2] and are either classified within the fungi or are closely related to them [3,4]. A common feature of microsporidian genomes is their significant reduction in size [5,6,7], reflecting their adaptation to intracellular parasitism at the molecular, cellular, and biochemical levels [8,9,10]. More than 1400 microsporidia from over 200 genera have been reported to date. As a genus of microsporidia, *Nosema* parasitizes a diverse array of insects and other arthropod hosts, leading to a disease known as nosemosis [11,12].

The most extensively studied *Nosema* species are *N. apis* and *N. ceranae*, which infect the European honey bee, *Apis mellifera*, and the Asian honey bee *Apis cerana*, respectively. These parasites invade the midgut and reproduce within intestinal cells, leading to various negative consequences, including damaged immune system barriers [13,14,15], reduced foraging activity, decreased colony strength, increased mortality, and shortened lifespans [16,17,18]. In the domesticated silkworm *Bombyx mori*, *N. bombycis*, another *Nosema* parasite, has caused significant reductions in silk production, resulting in substantial economic losses [19]. *Nosema* has also been found to infect other beneficial insects, including parasitoid wasps of the genus *Muscidifurax* (Hymenoptera: Pteromalidae), which serve as biological control agents for agricultural pest flies [20]. *N. muscidifuracis* infects the parasitoid wasp species *Muscidifurax zaraptor* and *M. raptor*, causing ~50% reduction in longevity and ~90% reduction in fecundity [21]. Understanding the evolutionary dynamics of *Nosema* parasites can inform the development of improved management and control strategies, potentially leading to positive impacts on ecosystem health, agricultural productivity, and global food security.

Since the pioneering genomic investigations into bee-infecting *Nosema* species, seven *Nosema* genomes have been assembled and annotated from a diverse range of hosts: the amphipod *Gammarus duebeni* [22], silk moth *Bombyx mori* [19], the Chinese tussar moth *Antheraea pernyi* [23], the cabbage butterfly *Pieris rapae* [24], the Asian honey bee *Apis cerana* [25,26,27], the European honey bee *Apis mellifera* [28], and, recently, our work on the microsporidium infecting the parasitoid wasp genus *Muscidifurax* [29]. The availability of these *Nosema* genomes enables comparative genomic analyses to investigate the evolutionary characteristics within the diverse *Nosema* genus.

In this study, we utilized the recently assembled *Nosema* genomes and conducted evolutionary and comparative genomic analyses. Building on findings from bee-infecting *Nosema* species, we investigated whether these results could be generalized across the entire genus. Our findings offer new insights into the evolution of telomeric repeat motifs, GC content, gene expression regulation, host switching, and codon usage bias across these genomes. The findings of this research could inform potential management strategies for nosemosis, particularly with regard to our discovery of a putative regulatory motif associated with the regulation of gene expression.

## 2. Materials and Methods

### 2.1. Source of Genome Data

The genome data analyzed in this study include the following genome assemblies (Table S1): *Encephalitozoon cuniculi* [8,30], *Nosema apis* BRL 01 [28], *Nosema muscidifuracis* [29], *Nosema ceranae* BRL 01 [25], *Nosema ceranae* PA08 [26], *Nosema ceranae* BRL [27], *Nosema bombycis* CQ1 [19], *Nosema granulosis* Ou3-Ou53 [22], *Nosema antheraeae* YY [23], and *Nosema* sp. YNPr [24].

### 2.2. Telomeric Repeat Identification

The TRIP (Telomeric Repeats Identification Pipeline) [31] was utilized to de novo predict the candidate telomeric repeat motifs (TRMs) from the publicly available short-read sequencing data of *Nosema* genomes (Table S2). With PacBio long-read assembly, the telomeric repeat motifs of *N. muscidifuracis* were identified from the repetitive regions at the termini of several contigs (Table S3). To confirm the telomeric repeats, we extracted and aligned the sequences of assembled telomeric and subtelomeric regions in the *N. muscidifuracis* genome (Supplementary Materials S1). A phylogenetic tree based on 27 nucleotide sequences of ~20 Kb subtelomeric regions was constructed to determine the evolutionary relationships of the conserved subtelomeric sequences (Supplementary Materials S2). *Encephalitozoon* TRMs were identified from long-read genome assemblies of *E. cuniculi* (NCBI Assembly accession number GCA_027571585), *E. hellem* (GCA_029215505), and *E. intestinalis* (GCA_024399295). At least 100 tandem repeat units are required at the chromosomal termini for TRM calls (Table S4).

### 2.3. Functional and Pathway Annotation of Nosema Muscidifuracis Proteins

The pathway annotation was performed using 2783 annotated genes in *N. muscidifuracis* and 5886 genes in *Saccharomyces cerevisiae* (*S. cerevisiae*, accession number: GCA_002571405.2) [32]. Assignments to genes in the metabolic and regulatory pathways were performed by the KEGG’s internal annotation tools (https://www.kegg.jp/, accessed on 4 March 2022) [33,34]. GhostKOALA was used to assign the most appropriate K numbers to the query genes by the GHOSTX program [35] and KOALA (KEGG Orthology And Links Annotation) algorithm [36], which is based on the sequence similarity search against the structured KEGG GENES database [37]. Subsequently, a set of K numbers was linked to KEGG pathway maps using the KEGG Mapper Reconstructed tool. The number of genes in selected KEGG pathways in *N. muscidifuracis* [29] and *S. cerevisiae* genomes were manually counted according to the KEGG pathway maps (Table S5 and Supplementary materials). Statistical significance was evaluated using the Chi-squared test.

### 2.4. Motif Discovery in 5′ Regulatory Regions in Nosema Muscidifuracis Genome

In microsporidia, the regulatory motifs of transcript initiation sites appear to be concealed in a short *cis*-regulatory region located upstream of the gene, a consequence of their compact and gene-dense genomes [38,39]. To identify the potential regulatory motifs in the 5′ context of *N. muscidifuracis* coding sequences, 200 bp sequences upstream of the start codon for all genes were extracted from the *N. muscidifuracis* genome. MEME version 5.4.1 [40] was applied to search for novel 5′ motifs using all gene sets (*n* = 2718) with the maximum motif width of 12 positions. To characterize the motif in *Nosema* species, the same analysis was performed in other *Nosema* genomes and *E. cuniculi*. We utilized MEME to identify the motifs located upstream of the start codon in a small, conserved gene set (n = 449 shared orthologous genes; Table S6 and Data S4) and other predicted genes in seven *Nosema* species and *E. cuniculi* (Table S7). The RNA-seq data described in our previous research were utilized to provide further evidence supporting the identified regulatory motifs. Average RNA-seq coverage across regions 200 bp upstream (−200 bp) and 500 bp downstream (+500 bp) was plotted for 2155 protein-coding genes in *N. muscidifuracis*. The presence of motifs in groups of genes with varying expression levels was analyzed to determine the relationship between predicted motifs and gene expression.

### 2.5. Phylogenetic Analyses

To infer the evolutionary history of *Nosema* genomes, we investigated the phylogenetic relationships between *N. muscidifuracis* and other microsporidian species. Homologous orthologs were determined for 10 strains across 7 *Nosema* species: *N. ceranae* BRL 01 [25], *N. ceranae* PA08 1199 [26], *N. ceranae* BRL [27], *N. apis* BRL 01 [28], *N. bombycis* CQ1 [19], *N. granulosis* Ou3-Ou53 [22], *N. antheraeae* YY [23], *Nosema* sp. YNPr [24], *N. muscidifuracis* Mzar, and *N. muscidifuracis* Mrap [29], along with the outgroup species *Encephalitozoon cuniculi* (*E. cuniculi*) [8,30]. The orthologs within the *Nosema* genus were extracted from OrthoDB v10.1 [41] using TaxonKit [42]. The genomic data of five *Nosema* strains and *E. cuniculi* were downloaded from NCBI (Table S1). The genomic data of *N. antheraeae* YY was downloaded from the SilkPathDB database [23], and the protein sequence of *Nosema* sp. YNPr was provided by Dr. Xu Jinshan via personal communication [24]. To identify the orthologs in these microsporidia genomes, a BLASTp search was conducted using the protein sequences from the *N. ceranae* (PA08 1199 strain) with a minimum of 20% sequence identity (E-value < 1 × 10^−5^). These orthologs in PA08 were subsequently validated using reciprocal best BLAST hits. A total of 449 orthologs among these eleven genomes were identified. Subsequently, MAFFT v7.407 [43] was utilized to align the protein sequences of orthologs among the above genomes (FFT-NS-2 algorithm). The protein alignments were concatenated into a single super-sequence (Data S5) to construct the phylogeny using the Jones–Taylor–Thornton (JTT) protein model with RAxML v8.2 [44]. A total of 1000 rapid bootstrap replicates were performed to evaluate the branch supports. The phylogenetic tree (Supplementary Materials) was visualized in FigTree v1.4.4 software (http://tree.bio.ed.ac.uk/software/figtree/, accessed on 10 November 2022). In addition, individual protein trees for the 449 orthologs were generated using the same approach as the concatenated tree, and the topology frequencies among these were compared to the concatenated topology. The topology and branch times of the host phylogeny were determined from published phylogenetic studies of the relevant taxa that combine fossil and molecular data for *Apis* [45], Hymenoptera [46], Lepidoptera [47,48], and Arthropoda [49].

### 2.6. GC Content Evolution and Codon Usage Bias

Considering the extremely low GC content in the *N. muscidifuracis* genome, we characterized the codon usage patterns of *N. muscidifuracis* genes. The codon usage of the protein-coding sequences was analyzed by CodonW software version 1.4.2 (http://codonw.sourceforge.net/, accessed on 15 October 2022) [50]. Various indices of codon usage bias were calculated, including the frequency of the synonymous codons at the third position of each base T, C, A, and G (T3s, C3s, A3s, and G3s), the average GC content of the first and second positions (GC12), GC content at the third position of synonymous codons (GC3s), GC content of the protein-coding gene (GC), the frequency of optimal codons (Fop), codon adaptation index (CAI), codon bias index (CBI), and the frequency of the synonymous codons for each amino acid. These calculations were systematically conducted across all *Nosema* species and *E. cuniculi* (Table S8 and Supplementary Materials). A comparative analysis of codon usage bias was performed to determine potential variations and similarities in the genetic coding preferences. To infer the ancestral states of the genome-wide G-C content in the *Nosema* genus, we employed a Bayesian approach using the random walk and MCMC models implemented in the software BayesTraits V4.1.3 [51]. A total of 2 million iterations were executed with a burn-in of 10,000, and every 11,000th iteration was sampled.

## 3. Results

### 3.1. Telomeric Repeat Motif (TRM) Characterization Revealed a Canonical TTAGG Motif and a Novel Composite TAGG/TTAGG Telomere

#### 3.1.1. TTAGG Is Likely to Be the Ancestral Form of TRM in *Nosema* and Microsporidian Species

The telomeric repeat motifs (TRMs) in the fungal parasite genus *Nosema* have not been specifically characterized or reported in the literature. To investigate telomere evolution within this genus, we performed de novo TRM prediction across multiple *Nosema* species (Table S1) based on short-read genome sequencing data (see Section 2). In *N. ceranae*, we identified TTAGG as the TRM candidate. For the remaining *Nosema* species with low sequencing depth, no TRM candidates were detected (Table S2). To determine if TTAGG is the typical TRM in microsporidia, we analyzed the chromosomal termini of publicly available long-read assemblies of three *Encephalitozoon* species, a microsporidian genus infecting vertebrate (see Section 2). All 11 chromosomes in *E. hellem* and *E. intestinalis*, as well as 10 out of 11 chromosomes in *E. cuniculi*, have TTAGG as the TRM (Table S3), suggesting that it is likely the ancestral form in microsporidia.

#### 3.1.2. A Novel Composition 4 bp/5 bp Form of TRM in *N. muscidifuracis*

**Figure 1:**
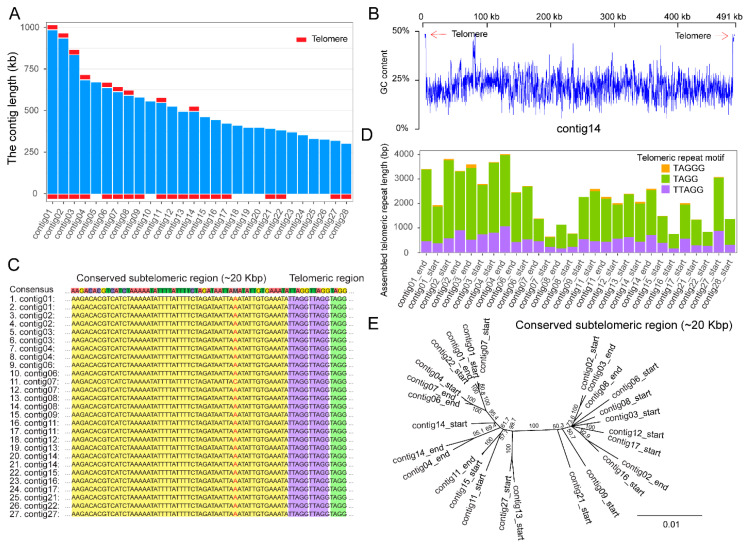
A novel type of telomere in the *Nosema Muscidifuracis* genome. (**A**) Presence of telomeric sequences at the termini of 28 *N. Muscidifuracis* genome contigs. (**B**) Plot of GC content along contig14 showing the high GC content at telomeric regions. (**C**) Sequence alignment at the telomere-subtelomere boundaries, showing the novel composite 4 bp and 5 bp telomeric repeat motifs. (**D**) Total length and relative abundance of telomeric repeat motifs (TAGG, TTAGG, and TAGGG) in telomeric regions. (**E**) Phylogenetic tree of 27 subtelomeric sequences from different genomic contigs in *N. muscidifuracis*. (Yellow shading, subtelomeric region. Red color, positions that are not identical across all contigs. Purple shading: TTAGG repeats in telomeric region. Green shading: TAGG repeats in telomeric region).

In *N. muscidifuracis*, a species that infects parasitoid wasps, two TRMs were identified: the canonical TTAGG and a novel 4 bp tandem repeat TAGG. The TAGG TRM is the most abundant, accounting for 75.3% of *N. muscidifuracis* telomeres (Table S4). The TAGG and TTAGG types of TRMs were detected at 19 chromosome ends among 28 contigs (Figure 1A and Data S1), exhibiting significantly higher GC content at the ends of chromosomes (Figure 1B). The 5 bp TTAGG motifs are interspersed among the predominant TAGG repeats (Figure 1C), forming a composite type of telomeric repeats. A third repeat type, TAGGG, is also present in the telomeric region, albeit at an extremely low abundance of 1.5% (Figure 1D).

#### 3.1.3. Two Major Types of Highly Conserved Subtelomeric Regions in *N. muscidifuracis*

When the *N. muscidifuracis* subtelomeric regions were characterized and aligned, we discovered that ~20 Kb subtelomeric regions immediately adjacent to the telomeric repeats are highly conserved (Figure 1C). Phylogenetic analysis revealed two major types of conserved subtelomeric sequences in *N. muscidifuracis* (Figure 1E and Supplementary Materials). These findings suggest diverse routes of telomere and subtelomere evolution in this species.

### 3.2. Extensive Genome Reduction in Nosema Highlights Loss of Mitochondrial Genes and Metabolic Pathways

#### 3.2.1. Retention of Certain Essential Biological Pathways Despite Severe Genome Reduction

**Figure 2:**
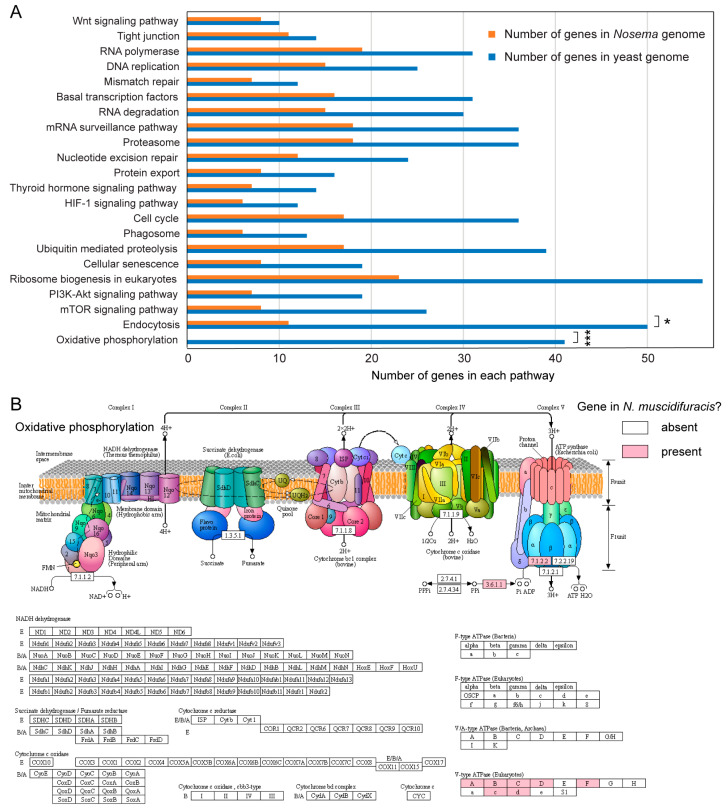
**Functional pathway specific genome reduction in *Nosema muscidifuracis*.** (**A**) Gene number in 23 pathways in *Nosema muscidifuracis* and *Saccharomyces cerevisiae* (Chi-squared test, *, *p* < 0.05; ***, *p* < 0.001). (**B**) KEGG pathway analysis of *Nosema muscidifuracis* mitochondrial proteins suggested that the entire electron transport chain and eukaryotic F-type ATPase were completely missing in the mitochondrial oxidative phosphorylation metabolic pathway. The enzymes/proteins that are present in the *N. muscidifuracis* genome are shaded in red.

By analyzing multiple *Nosema* species, we conducted a more detailed exploration of genome reduction processes within this genus than what was reported in previous literature. Compared to the genome of the free-living yeast *Saccharomyces cerevisiae* [52,53], *Nosema* species only possess about one-third to one-half of the genes. This significant reduction indicates that *Nosema* has undergone extensive genome contraction during its evolution. The reduction in genome size is presumably achieved through the loss of genes involved in metabolic pathways and cellular processes that are no longer essential for survival. Despite a significant genome reduction, functional annotation of *N. muscidifuracis* proteins revealed that more than 50% of the genes in several critical pathways are preserved (Table S5 and Supplementary Materials). These pathways include DNA replication, mismatch repair, tight junction formation, RNA polymerase function, Wnt signaling, and basal transcription factors (Figure 2A). The retention of these pathways suggests that, even with a significantly reduced genome, *Nosema* species maintain essential cellular functions necessary for their survival and replication within host cells.

#### 3.2.2. Significant Gene Loss in Oxidative Phosphorylation Pathway Coincides with the Absence of Mitochondrial Genes and the Organelle

A notable genomic feature of microsporidia is the absence of mitochondria, which have been transformed into highly modified and reduced organelles known as mitosomes [54]. In *N. muscidifuracis*, we observed a significant loss of genes associated with the oxidative phosphorylation pathway (0 in *Nosema* and 41 in yeast; Figure 2B), which aligns with the complete absence of mitochondrial genes and the organelle itself. Genes encoding the mitochondrial electron transport complexes, whether typically located in the mitochondrial or nuclear genomes, were completely absent (*p* < 0.001, Chi-squared test; Figure 2B). Furthermore, the mitochondrial F-type ATPase was absent from the oxidative phosphorylation metabolic pathway (Figure 2B). The lack of these genes indicates that *Nosema* species have entirely lost the capability for mitochondrial respiration and ATP production through this pathway.

#### 3.2.3. Significant Reduction in the Endocytosis Pathways

Endocytosis represents another pathway that exhibits a significant reduction in the number of genes, with 11 genes identified in *Nosema* compared to 50 in yeast (*p* < 0.05, Chi-squared test; Figure 2 and Figure S1). With a limited number of genes associated with endocytosis, *Nosema* has a reduced ability to independently acquire certain molecules, resulting in a heavy reliance on absorbing nutrients directly from host cell cytoplasm. On the other hand, by expressing fewer transmembrane proteins, *Nosema* may utilize the host’s endocytic pathways, and avoid triggering the host’s innate immune defense mechanisms.

### 3.3. A Conserved Regulatory Motif Upstream of Nosema Protein-Coding Genes

#### 3.3.1. Discovery of a Highly Over-Represented 12 bp Motif in *N. muscidifuracis*

**Figure 3:**
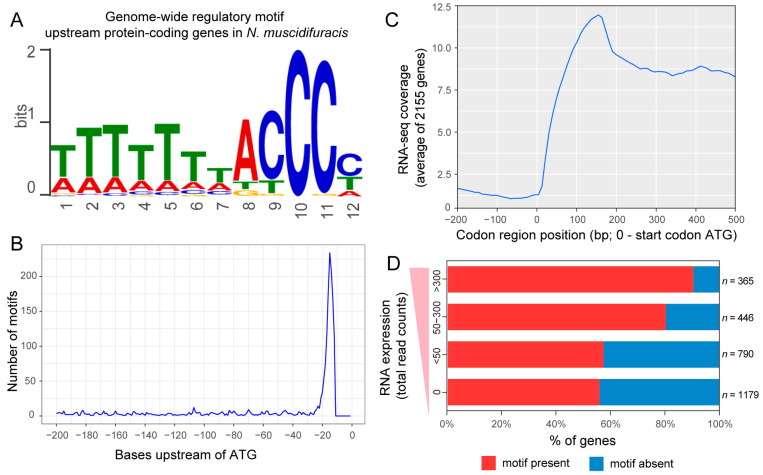
A motif associated with translation start sites and gene expression levels in *Nosema muscidifuracis*. (**A**) A sequence motif enriched upstream of *N. muscidifuracis* genes, containing a homopolymer of seven thymine (T) nucleotides, followed by an adenine (**A**) and three consecutive cytosine (C) nucleotides. (**B**) Distribution of the motif upstream of the gene regions. The *x*-axis measures the distance from the first nucleotide of the motif to the start codon in bases, and the *y*-axis indicates the number of detected motifs. (**C**) Average RNA-seq coverage across protein-coding gene regions in *N. muscidifuracis*. (**D**) The percentage of genes with the motif in gene groups with different expression levels.

Previous studies on bee-infecting *Nosema* species have identified potential regulatory motifs containing the cytosine triplets TTTTTTTACCCC [25] and ACCCTT [28]. Through motif prediction in the 5′ regulatory regions of *N. muscidifuracis* (see Section 2), we discovered that a single 12-base pair motif, TTTTTTTACCCC, is highly overrepresented (E-value = 1.6 × 10^−256^; Figure 3A). Within this motif, the sequence consisting of a single adenine followed by a cytosine quadruplet (ACCCC) was highly significant, occurring much more frequently than would be expected by chance (*p* < 0.001). Interestingly, genome distribution analyses revealed that the 2712 occurrences of this motif are predominantly located within 20 base pairs upstream of the start codon of protein-coding genes. (Figure 3B).

#### 3.3.2. Positive Association of the Presence of TTTTTTTACCCC Motif with Gene Expression

Although the C-rich motif was previously suspected to regulate gene expression in earlier studies, the actual relationship between the motif and gene expression has yet to be clarified. RNA-seq coverage analyses revealed that the TTTTTTTACCCC motif is located immediately upstream of expressed transcripts in *N. muscidifuracis* (Figure 3C). To assess the relationship between this motif and gene expression levels, we analyzed its prevalence in groups of genes categorized as highly, moderately, and lowly expressed. Our findings showed that more than 90% of the highly expressed genes contain the TTTTTTTACCCC motif, whereas only about 55% of non-expressed genes have this motif (*p* < 0.05, Chi-squared test; Figure 3D). This association between the presence of the motif and higher gene expression levels supports the hypothesis that TTTTTTTACCCC is a candidate regulatory motif for active gene expression.

#### 3.3.3. Conservation of CCC-Containing Motif in *Nosema* and Microsporidian Species

**Figure 4:**
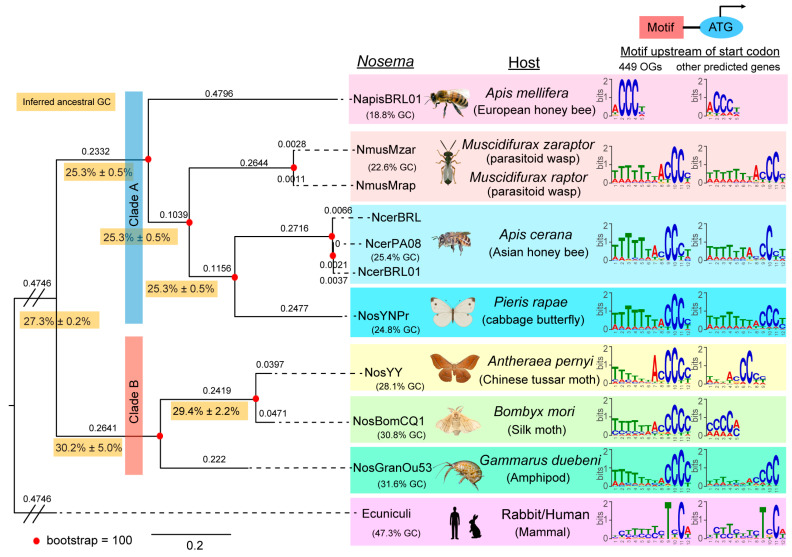
Phylogenomic analysis revealed a host switch event and conserved sequence motifs in *Nosema*. A maximum-likelihood tree of *N. muscidifuracis* isolated in parasitoid wasps *Muscidifurax zaraptor* (NosMusMzar) and *M. raptor* (NosMusMrap) with other *Nosema* was constructed based on 449 shared proteins. The *Nosema* species/strains included are *N. apis* strain BRL01 (NosApis), *N. ceranae* strain PA08 1199 (NcerPA08), *N. ceranae* strain BRL (NcerBRL), *N. ceranae* strain BRL01 (NcerBRL01), the tussar moth *Antheraea pernyi Nosema strain YNPr* (NosYNPr), *N. antheraeae* strain YY (NosYY), *N. bombycis* strain CQ1 (NosBomCQ1), and *N. granulosis* strain Ou3-Ou53 (NosGranOu53). The *Encephalitozoon cuniculi* GB-M1 strain (Ecuniculi) was included as the outgroup. The bootstrap value is indicated by dots, with red representing a support level of 100/100. The length of each branch is indicated beneath the branches. The sequence logos displayed the conserved motifs located upstream of the start codons, as predicted by MEME using 449 shared orthologous genes and other gene models in the seven *Nosema* species and *E. cuniculi*. Genome-wide average G-C content for each species is displayed beneath their respective logos. The inferred ancestral G-C content, along with the standard deviation, is labeled near the nodes and shaded in orange.

To determine whether the TTTTTTTACCCC motif is also present in other *Nosema* and microsporidian species, we conducted a de novo search of 5′ motifs in six additional *Nosema* genomes and *Encephalitozoon cuniculi* (Table S1) in 449 shared orthologous genes (Data S4). Using the same parameters as in our analysis of *N. muscidifuracis*, we identified a shared motif pattern. Notably, all seven *Nosema* species’ motifs contain a 3 bp or 4 bp cytosine homopolymer core (CCC or CCCC; Figure 4), which is extremely rare in A-T-rich *Nosema* genomes (Figure 4). In addition, six of the seven *Nosema* species have a leading thymine stretch (TTTTTT or TTTTTTT), whereas *Nosema apis* lacks this feature (Figure 4). When we performed the same analysis using non-orthologous predicted genes, the conserved CCC/CCCC cores were still identifiable within the significant motifs (Figure 4 and Table S7). Such C homopolymers were not detected in the outgroup species *E. cuniculi* (Figure 4). Our findings indicate that the cytosine-rich motif is conserved across the *Nosema* genus, reinforcing its potential role in the regulation of gene expression.

### 3.4. Phylogenomic Analysis of Microsporidian Genomes Revealed Nosema Host Switch Events

We constructed a phylogenetic tree using the sequences of 449 orthologous protein-coding genes from *Nosema* genomes (Table S1 and Supplementary Materials), with *E. cuniculi* serving as an outgroup to investigate their evolutionary relationships (Figure 4 and Supplementary Materials). As expected, the three strains of *N. ceranae* clustered together on the same branch, and the two strains of *N. muscidifuracis* were closely related (Figure 4). Interestingly, there are two major groups, Clade A and Clade B, which are supported by strong (100%) bootstrap values. Further support of the validity of these two clades is that they differ in their genome-wide GC content (20–25% GC for Clade A and 28–32% for Clade B; see Section 3.5). However, the topology of the *Nosema* phylogenetic tree is not completely congruent with the host phylogeny. Most notably, the cabbage butterfly (*Pieris rapae*) *Nosema* is most closely related to the *Nosema* found in the bee *Apis cerana*, and this association has 100% bootstrap support (Figure 4). Furthermore, the cabbage butterfly *Nosema* is embedded in Clade A (Figure 4) with four hymenopteran *Nosema* (*A. mellifera*, *Muscidifurax zaraptor*, *M. raptor*, and *A. cerana*), and this clade has 100% bootstrap support, whereas the other two *Nosema* associated with Lepidoptera (*Bombyx mori* and *Antheraea pernyi*) occur in a separate strongly supported Clade B (100% bootstrap value, Figure 4), and are more closely related to a *Nosema* found in crustaceans (again with 100% support).

Analyses of individual protein trees further support the cabbage butterfly host shift. A total of 444 proteins (98.9%) cluster the cabbage butterfly *Nosema* with the hymenopteran *Nosema* strains, whereas only 3 (0.7%) join it with the other two lepidopterans. Therefore, the data strongly support a host shift involving the *P. rapae Nosema*. The most likely direction of this shift is from a hymenopteran *Nosema* into the butterfly, although directionality cannot be resolved at this time. Two lepidopteran *Nosema* are found in the moths (*An. pernyi* and *B. mori*), and these cluster together in Clade B with 100% bootstrap support. Their hosts belong to the superfamily Bombycoidea, whereas *P. rapae* is in the superfamily Papilionoidea, with an estimated divergence time from the two moths of 100 million years [47]. Interestingly, *B. mori* and *An. pernyi* form a group with the crustacean *Gammarus duebeni Nosema* with 100% bootstrap support, whereas the Hexapoda (including insects) and Crustacea are estimated to have diverged 506 million years ago [49]. There are likely to be additional host shifts involved here, although there are insufficient numbers of well-assembled *Nosema* genomes to resolve the details at this time.

Finally, a host shift involving the two *Nosema* found in the bees *A. mellifera* and *A. cerana* is likely. The bootstrap value for the clade combining *A. cerana Nosema* with the strains found in the two parasitoid wasp species *M. zaraptor* and *M. raptor* is strongly supported (100%), with the second *Apis Nosema* found in *A. meliferra* as an outgroup. The parasitic wasps belong to the Chalcidoids, which diverged from the lineages leading to honey bees approximately 247 MYA [46], whereas the two *Apis* species are estimated to have diverged only 23.7 MYA [45]. To exclude the possibility of potential artifacts caused by concatenation, we examined the 449 individual gene trees. Of these, 89.5% (402/449) of *A. cerana Nosema* have *Muscidifurax* as their closest hymenopteran relative, whereas only 10.5% have the host congener *Apis mellifera* as the closest hymenopteran neighbor. This phylogenetic incongruency indicates that *Nosema* parasites have likely moved between bee and wasp hosts at least once in the past; however, the direction of this transfer is not yet resolved.

The two *Apis Nosema* grouped together relative to the parasitoid *Nosema* for 12 individual proteins. They may represent genetic exchanges of the *Nosema* with shared *Apis* hosts, or convergent evolution. Here, we highlight protein OG398, whose closest BLAST hit is the *Myg1*-like protein in *E. cuniculi* (88% sequence identity, E-value = 3 × 10^−18^). *Myg1* encodes a 3′-5′ RNA exonuclease regulating the spatial segregation of organellar RNA processing and serving as a coordinator of nuclear-mitochondrial translational crosstalk [55]. It is involved in ribosome assembly and cytoplasmic translation by processing pre-rRNA, and it modifies the 3′ ends of mitochondrial rRNA (mito-rRNA) and messenger RNA (mRNA), thereby influencing mitochondrial translation. Based on protein sequence homology, we speculate that OG398 may play a role in hijacking and rebalancing the host’s mitochondrial translation to meet their own energy needs. Further studies of these and similar discordant protein topologies may be warranted in the future, especially once more *Nosema* genome assemblies are available.

### 3.5. Evolution of GENOME-Wide G-C Content and Codon Usage Bias

#### 3.5.1. Potential Directional Shift from GC-Rich to AT-Rich Genomes in *Nosema* Evolution

Microsporidian pathogens infecting mammals, such as *E. cuniculi*, have a high G-C content with nearly 50% GC [8]. Similarly, *Nematocida displodere*, a microsporidian parasite of *Caenorhabditis elegans*, has a genome-wide G-C content of 49.2% [56]. Another microsporidian species, *Antonospora locustae*, which infects locusts, has an average genome G-C content of 42.0% [57], suggesting many microsporidia have relatively high G-C content. In contrast, the published genomes of *Nosema* species have reported much lower G-C contents, ranging from 25% to 35%. Based on the *Nosema* phylogeny, we inferred the ancestral states of G-C content on the nodes and found a significant reduction in genome-wide G-C content in *Nosema muscidifuracis* and *Nosema apis* (Figure 4), suggesting a tendency of decreasing G-C content during *Nosema* evolution.

#### 3.5.2. Significant Codon Bias in A-T Rich *Nosema* Genomes

Since the *Nosema* genomes are gene-dense, a reduction in G-C content significantly impacts their protein-coding genes. We examined the G-C content at the third codon position (GC3) and the first two codon positions (GC12) in three species: *N. muscidifuracis*, *N. ceranae*, and *E. cuniculi*. Our analysis revealed that *E. cuniculi* has an 11.6% higher GC12 compared to the two *Nosema* species (43.7% vs. 32.1%). Moreover, the GC3 in *E. cuniculi* is significantly higher at 40%, which distinctly separates its genes from those of *Nosema* (Figure S3). These results are consistent with what was reported in a previous study [25].

**Figure 5:**
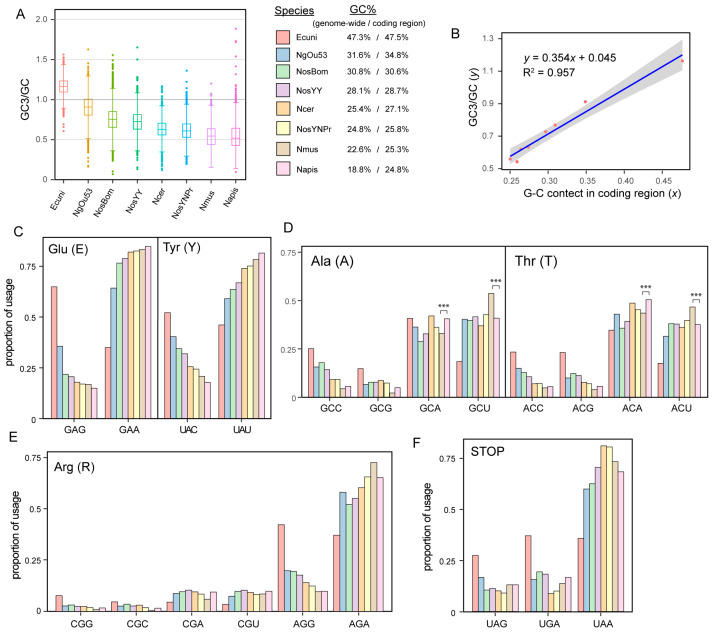
Codon bias and evolution toward AT-rich genomes in *Nosema*. (**A**) Boxplot of GC3 (G-C content at the 3rd codon position) over G-C content at all condo positions, rank ordered by the genome average G-C content in *Encephalitozoon cuniculi* (Ecuni), *Nosema granulosis* (NgOu53), *Nosema bombycis* (NosBom), *Nosema antheraeae* (NosYY), *Nosema ceranae* (Ncer), *Nosema* sp. *YNPr* (NosYNPr), *Nosema muscidifuracis* (Nmus), and *Nosema apis* (Napis). (**B**) The correlation between coding region G-C content (*x*-axis) and GC3/GC (*y*-axis). (**C**) Proportion of codon usage for glutamic acid and tyrosine in eight microsporidian genomes. The proportion of arginine codon usage across eight microsporidian genomes. (**D**) Proportion of codon usage for alanine and threonine in eight microsporidian genomes (Chi-squared test, ***, *p* < 0.001). (**E**) Proportion of codon usage for arginine in eight microsporidian genomes. (**F**) Proportion of STOP codon usage in eight microsporidian genomes.

*N. muscidifuracis* exhibits a significantly lower G-C content (22.6%) compared to *N. ceranae* (25.4%). The GC12 values are comparable between *N. muscidifuracis* (31.9%) and *N. ceranae* (32.4%), which may reflect the selective constraints at the first two codon positions. However, the GC3 in *N. muscidifuracis* (14.1%) is significantly lower than that in *N. ceranae* (17.3%), which may contribute to the differences observed in their overall nucleotide composition (Figure 5A). When all seven *Nosema* species are compared, those with lower overall G-C content tend to exhibit lower GC3/GC ratios (Figure 5A). There is a linear relationship between GC3 and total GC (R^2^ = 0.957; Figure 5B), indicating that low-GC *Nosema* species prefer to use codons that end with A or T. This preference indicates a codon usage bias toward A/T at the third codon position in species with lower G-C content.

#### 3.5.3. Species-Specific Usage Preference of Degenerative Codons

When analyzing degenerative codons, we discovered an overall codon bias toward the A/T at the third position (Figure S4), with the degree of bias closely correlated with the genome-wide G-C content (Figure 5C). However, we also observed differential preferences for A or T at four-fold degenerate sites in each *Nosema* species. For example, the codons for alanine and threonine are significantly more biased toward uracil (U) at the third position in *N. muscidifuracis* (with a G-C content of 31.9%), while a larger proportion of codons with an A at the third position is observed in *N. apis* (*p* < 0.001; Figure 5D).

#### 3.5.4. CpG Avoidance in Arginine Encoding Codons

Arginine is encoded by six codons, four of which begin with the CpG dinucleotide. When comparing the usage of CGA/CGU codons to AGG codons, which have the same G-C content, we observed that AGG is overrepresented in five *Nosema* species and in *E. cuniculi* (Figure 5E). This preference indicates a tendency to avoid the CpG dinucleotide context in arginine codons. The depletion of CpG sites is presumably due to spontaneous deamination processes occurring in the genome. In this process, methylated cytosines can deaminate to thymine, leading to mutations that reduce the frequency of CpG dinucleotides over time.

#### 3.5.5. Usage Bias of STOP Codons in *Nosema* Species Compared to *Encephalitozoon*

A usage bias of stop codons was observed in *Nosema* species. Specifically, among the three stop codons (UAG, UGA, and UAA), there appears to be no preference in *E. cuniculi* (Figure 5F). In contrast, the low G-C content *Nosema* species exhibit varying degrees of bias toward using UAA as the stop codon (Figure 5F). This suggests that *Nosema* species with lower genome-wide G-C content preferentially utilize UAA to terminate protein synthesis, whereas such STOP codon usage bias is not present in *E. cuniculi*.

## 4. Discussion

### 4.1. Discovery of Host Shift Events in Nosema Species

Host shifts are prevalent in microsporidia, and all four clades of microsporidia contain species that infect both insects and humans [3]. Host shifts frequently disrupt co-phylogenetic patterns between parasites and their hosts, leading to incongruences in their evolutionary trees [58]. Studies of microsporidian parasites *Dictyocoela roeselum* and *Nosema granulosis* in amphipods revealed coevolution and co-diversification with host species, whereas *Dictyocoela muelleri* and three species of *Cucumispora* showed recent colonization, indicating recent spreading and potential host shift [59,60]. Typically, host switch events occur among phylogenetically related hosts rather than between distantly related ones [61]. Until now, no significant host shift event had been identified within the *Nosema* genus. In this study, we discovered potential host shift events involving *Nosema* species infecting bees and wasps through phylogenomic analysis of multiple *Nosema* genomes. This finding suggests that host switching between distantly related host taxa can occur, serving as a potential mechanism for *Nosema* diversification. After transitioning to a different host, genetic adaptations might be necessary to overcome challenges related to host recognition, energy and metabolite utilization, immune tolerance, transmission, and proliferation in a new environment. We identified a few genes that may have undergone co-evolution following a host switch, including *Myg1*, which is involved in nuclear-mitochondrial translational crosstalk. Determining the gain and loss of specific genes remains challenging due to the insufficient quality of several available *Nosema* genomes. This highlights the need for improved genome assemblies in future research to gain a deeper understanding of the genetic adaptations associated with host shifts.

### 4.2. CCC-Containing Motifs Are Conserved in Multiple Nosema Species

One significant finding of this study is the conservation of CCC motifs across various *Nosema* species and their association with active gene transcription. Although CCC motifs have been previously reported in bee-infecting *Nosema*, *Encephalitozoon*, and *Antonospora* [30], our study confirms their broader presence within the *Nosema* genus and reveals a direct association with gene expression. A total of three different motifs were found to be present upstream of the microsporidian gene models: the AAATTT-like signal, the CCC-like signal, and the GGG-like signal [30]. Among these identified motifs, AAATTT and GGG were only found in selected ribosomal genes using 5′ RACE, and we did not discover any of these motifs reaching statistical significance in *Nosema* species.

CCC-like signal-containing motifs have been identified in two bee-infecting *Nosema* species. Specifically, the motif TTTTTTTACCCC was identified in *N. ceranae* [25], and the ACCCTT motif has been reported in *N. apis* [28]. The *N. ceranae* study also identified a CCC motif in *E. cuniculi* genes, but it did not reach statistical significance [25]. Our study did not find any CCC-containing motifs in *E. cuniculi*, which is consistent with the lack of statistical significance observed in the literature [25]. We did identify such CCC motifs in *N. ceranae* (TTTTTTTACCCC, identical to [25]) and *N. apis* (ACCCT, one bp shorter compared to [51]), thereby confirming previous findings. In addition, we discovered significant CCC-containing motifs in all five other *Nosema* species examined (*N. ceranae*, *N. bombycis*, *N. granulosis*, *N. antheraeae*, and *Nosema* sp. YNPr), indicating that the CCC motif serves as a key regulatory signal throughout the entire genus. This finding highlights the importance of CCC motifs beyond just bee-infecting *Nosema* species, suggesting a fundamental role in gene regulation across diverse *Nosema* hosts.

### 4.3. Association Between CCC-Containing Motifs and Active Transcription Suggests a Direct Role in Gene Regulation

Previous studies have demonstrated that CCC-containing motifs are located immediately upstream of the coding start site, suggesting a potential role in regulating gene expression [30]. In this study, we found that these CCC-containing motifs are located within 20 bp upstream of the start codon, demarketing the translation initiation site. Our RNA-seq data further demonstrated, for the first time, that these motifs are positively correlated with active transcription, supporting their role as cis-regulatory elements in gene expression regulation. The exact mechanism of their function, however, requires further investigation.

### 4.4. Evolution of CCC-Containing Motifs in Relation to Genome-Wide G-C Content in Nosema Species

Although CCC-containing motifs are ubiquitously present in *Nosema* species, we discovered variation in the number of consecutive Cs across the two *Nosema* clades. Given the generally AT-rich nature of *Nosema* genomes, homopolymers of C are unlikely to occur by chance. In *Nosema* clade A, which has a lower genome-wide G-C content (ranging from 18.8% to 24.8%), all motifs contain three consecutive Cs with information content > 0.5 bit. In Clade B, where G-C content is higher (28.1% to 31.6%), the motifs contain four consecutive Cs (CCCC). We hypothesize that the ancestral motif core was CCCC, which evolved into CCC in clade A as the G-C content decreased. This suggests that the evolution of motif cores may be linked to genome-wide G-C content, indicating coordinated evolution between specific regulatory motifs and overall G-C composition in *Nosema* lineages. Additional well-assembled and annotated *Nosema* genomes are needed to further test this hypothesis.

### 4.5. Insights into Telomere Evolution in Nosema and Microsporadia

Previous studies have shown that telomeric repeat motifs (TRMs) in most species follow the classical pattern of TxAyGz, such as TAGG, TTAGG, TTAGGG, and TTTAGGG [62]. In this study, we examined three published *Encephalitozoon* genomes with chromosome-level assemblies and found that TTAGG is the conserved TRM, which matches the canonical TRM found in insect hosts [63]. In *N. ceranae*, TTAGG has also been identified as the TRM candidate, suggesting that TTAGG might be the shared TRM among microsporidians.

In *N. muscidifuracis*, we discovered TTAGG repeats at the chromosomal termini; however, these repeats accounted for only approximately 20% of the telomeric regions. Instead, a variant that is one base pair shorter, TAGG, is the predominant TRM form found in the telomeres of this species. Based on phylogenetic parsimony, we propose that the TTAGG repeat is the ancestral form, while the TAGG repeat is a derived variant that has become dominant in the telomeres of *N. muscidifuracis*. This novel composite form of four-base-pair (TAGG) and five-base-pair (TTAGG) repeats has not been reported in any other microsporidian genomes, providing new insights into telomere evolution.

The exceptional completeness of the *N. muscidifuracis* genome enabled a comprehensive analysis of its subtelomeric regions. These regions were found to be highly conserved and form two distinct clades, suggesting that they may have different evolutionary histories. Further investigations are needed to understand how the composite TRM is maintained and how subtelomeric regions have evolved in *N. muscidifuracis* and other *Nosema* species.

### 4.6. Directional AT-Rich Evolution of Nosema Genomes

Significant variation in genome-wide G-C content has been observed among fungal species [64]. The evolution of G-C content is influenced by various evolutionary forces, including natural selection, constraints imposed by the host environment, population bottlenecks, random genetic drift, and others [65,66,67]. The vertebrate microsporidian *Encephalitozoon cuniculi* has nearly 50% G-C content, exceeding that of its host genomes. In contrast, *Nosema* species are typically AT-rich, exhibiting lower G-C content compared to their host genomes, consistent with previous reports on bee-infecting *Nosema* species.

Using ancestral state inference through a Bayesian approach, we discovered a significant reduction in G-C content in *N. apis* and *N. muscidifuracis* within the bee–wasp–butterfly *Nosema* clade. Among the species we examined, *N. granulosis* has the highest G-C content (32%) and infects the amphipod *Gammarus*, which first appeared in the Cretaceous period [68]. In contrast, *Nosema apis*, with the lowest G-C content (18.8%), infects eusocial bees that began to diversify tens of millions of years later in the Paleogene period [46]. Although the exact timing of *Nosema* infection acquisition in these host species remains uncertain, our findings suggest an evolutionary trajectory toward lower G-C content in these parasites.

The AT-rich nature of the *Nosema* genome may confer an advantage by allowing the parasite to utilize the more abundant ATP and TTP nucleotides available in host cells for their own genome replication and reproduction. Given that *Nosema* species lack nucleotide synthesis pathways and rely entirely on host metabolites for DNA replication [69], utilizing these readily available nucleotides could enhance their replication efficiency. However, the exact evolutionary forces driving this shift toward an AT-rich genome remain unclear and require further investigation.

### 4.7. Systematic Analysis of Codon Usage Revealed Common and Species-Specific Patterns in Nosema

Codon usage bias has been studied in *Nosema* and other microsporidian species [24,25,27,28]. We conducted a systematic analysis of codon bias across *Nosema* species and identified several common features. All *Nosema* species exhibit a global bias toward AT-rich codons. Specifically, the G-C content at the third codon position (GC3) is significantly lower than at the first two codon positions (GC12), and this reduction correlates well with the species’ overall G-C average across many codon positions. This suggests that the evolutionary trend toward AT-rich genomes in *Nosema* is achieved, at least in part, through G/C-to-A/T synonymous substitutions at the third codon positions. Additionally, we observed CpG avoidance in arginine codons and a bias toward using the AT-rich UAA stop codon in all *Nosema* species.

Despite overall similarities in the codon usage bias toward AT-rich codons among *Nosema* species, the specific patterns are not identical across all species examined. In *N. apis*, studies have identified a significantly higher G-C content in coding regions (25%) compared to the genome average (18.8%) [28]. Similarly, *N. muscidifuracis* shows a genome-wide G-C content of 22.6% versus 25.3% in its coding regions, as found in this study. However, this disparity between coding and genome-wide G-C content does not hold in *N. bombycis* or *N. antheraeae*, where the G-C content is similar in both coding and intergenic regions. These findings suggest varying levels of G-C reduction in coding versus intergenic regions among different *Nosema* species. In addition, the degree of codon bias is not always correlated with the genome-wide G-C content. For instance, for the codons encoding alanine (GCA/U) and threonine (ACA/U), *N. apis* prefers codons ending with adenine (A), whereas *N. muscidifuracis* favors those ending with uracil (U). This indicates the presence of species-specific preferences, which may reflect adaptations or evolutionary histories unique to each species.

### 4.8. Implications of Severe Genome Reduction and Lack of Mitochondria in Nosema

*Monocercomonoides* is the first known eukaryote lacking mitochondria [70], and in microsporidia, mitochondria have deteriorated into numerous tiny structures known as mitosomes [71]. These mitosomes have limited metabolic functions and are involved in processes such as iron–sulfur cluster assembly and lipid metabolism [72]. Microsporidia are derived from lineages that originally contained mitochondria, indicating that the loss of mitochondria is a secondary evolutionary event [70,73,74,75,76]. From our analysis of *N. muscidifuracis*, we found that mitochondrial rRNA, tRNA, and protein-coding genes are absent, and genes involved in the complexes of the electron transport chain are completely missing. This suggests that *Nosema* species may take advantage of host metabolism to survive as intracellular parasites [77].

Coupled with the loss of mitochondria, *Nosema* species exhibit severe genome reduction [78,79,80,81], presumably due to their highly specialized morphology and life cycle [9,82]. Endocytosis was identified as another major pathway experiencing significant gene loss in *Nosema* in this research. Previous studies have shown that microsporidian cells can exploit the host endocytosis pathways to exit intestine cells for spreading [83,84,85], reducing their need for genes related to this process compared to free-living fungi. This reduction highlights the extensive genome contraction these parasites have undergone as they adapt to an intracellular lifestyle. The loss of endocytosis-related genes suggests that Nosema species rely heavily on their hosts for nutrient uptake and cellular processes, further emphasizing their dependence on host metabolism for survival and proliferation. The selective gene loss indicates an evolutionary strategy where non-essential genes are lost, while vital processes are conserved to support their intracellular parasitic lifestyle. By analyzing more *Nosema* species, we can gain a more detailed understanding of which pathways are critical and how genome reduction has shaped their evolution.

## 5. Conclusions

In this study, our phylogenomic analyses of genome-available *Nosema* species uncovered incongruences between the *Nosema* tree and their host phylogenies, revealing host shift events involving butterflies, bees, and wasps. We found that TTAGG is likely the ancestral telomeric repeat motif in *Nosema* and other microsporidians; however, evolutionary fluidity exists in certain *Nosema* species that possess composite TTAGG/TAGG repeats. Additionally, cytosine-rich motifs upstream of protein-coding genes, linked to high gene expression, indicate a potential cis-regulatory function. Our findings expand on previous *Nosema* genome studies by shedding light on gene regulation, genome reduction, telomere evolution, host shifts, and codon usage bias. These insights have practical applications for managing nosemosis by targeting conserved regulatory motifs. Future research should further investigate the functions of these motifs, enhance genome completeness, and examine genetic adaptations to host shifts, supporting efforts to control these parasites and protect vital insect populations.

## Data Availability

The raw RNA-seq data and Nosema gene read counts are available at NCBI GEO (Gene Expression Omnibus) databases under the accession number GSE248484. Supplemental Data S1–S7 can be accessed through the Auburn University Scholarly Repository via the following link: http://dx.doi.org/10.35099/aurora-699.

## Supplementary Materials

The following supporting information can be downloaded at: https://www.mdpi.com/article/10.3390/biology13110952/s1, Table S1. Accessions for genome assemblies used in this study. Table S2. Short-read genome sequencing data in *Nosema* species used for telomeric repeat motif identification. Table S3. *Encephalitozoon* telomeric repeat motifs inferred from long-read genome assemblies. Table S4. Summary of the TRM call and composition of TAGG, TTAGG, and TAGGG repeat units in the telomere of *Nosema muscidifuracis*. Table S5. The number of genes in major functional pathways identified in *Nosema muscidifuracis* and *Saccharomyces cerevisiae.* Table S6. Motif sequences and significance identified in 449 shared orthologous genes in seven *Nosema* species and an outgroup species *Encephalitozoon cuniculi*. Table S7. Motif sequences and significance identified in predicted genes of seven *Nosema* species and *Encephalitozoon cuniculi*, excluding 449 shared orthologous genes. Table S8. Summary of codon usage in *Encephalitozoon cuniculi* and seven *Nosema* species. Figure S1. Presence and absence of genes in the endocytosis pathway in *Nosema muscidifuracis* and *Saccharomyces cerevisiae*. Figure S2. A maximum-likelihood tree of *Myg1-like* gene in seven *Nosema* genomes and the outgroup *Encephalitozoon cuniculi*. Figure S3. G-C content analysis of protein genes in *N. muscidifuracis*, *N. ceranae*, and *E. cuniculi*. Figure S4. Proportion of codon usage in seven *Nosema* species and *Encephalitozoon cuniculi*.
