# Supplementary material for: Genome Structure, Evolution, and Host Shift of Nosema
Source: Biology (Basel). 2024 Nov 19;13(11):952. doi: 10.3390/biology13110952 (PMC11592040; doi:10.3390/biology13110952)
Supplement: Supplementary file 1 [file biology-13-00952-s001.zip › FigureS1-S4_Nosema_genome_evolution_20231204.pdf]

Figure S1. Presence and absence of genes in the endocytosis pathway in *Nosema muscidifuracis* and *Saccharomyces cerevisiae*.

KEGG endocytosis pathway showing the presence and absence of genes in *Nosema muscidifuracis* and *Saccharomyces cerevisiae*.

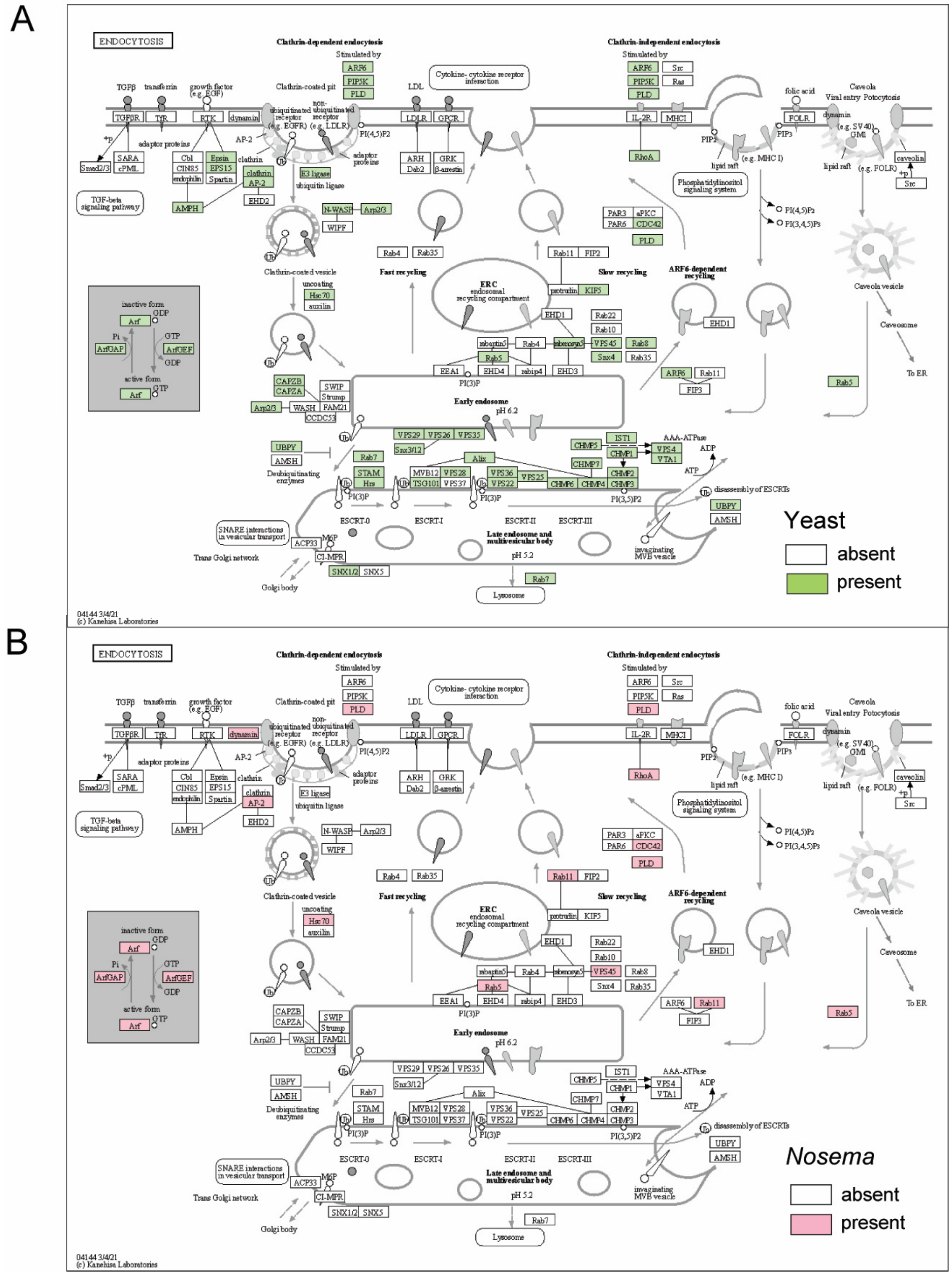

Figure S2. A maximum-likelihood tree of *Myg1-like* gene in seven *Nosema* genomes and the outgroup *Encephalitozoon cuniculi*.

The length of each branch is shown under the branches. Bootstrap values are color-coded at the branch points: red, 100/100 support, orange, 95/100 support, blue, 80/100 support.

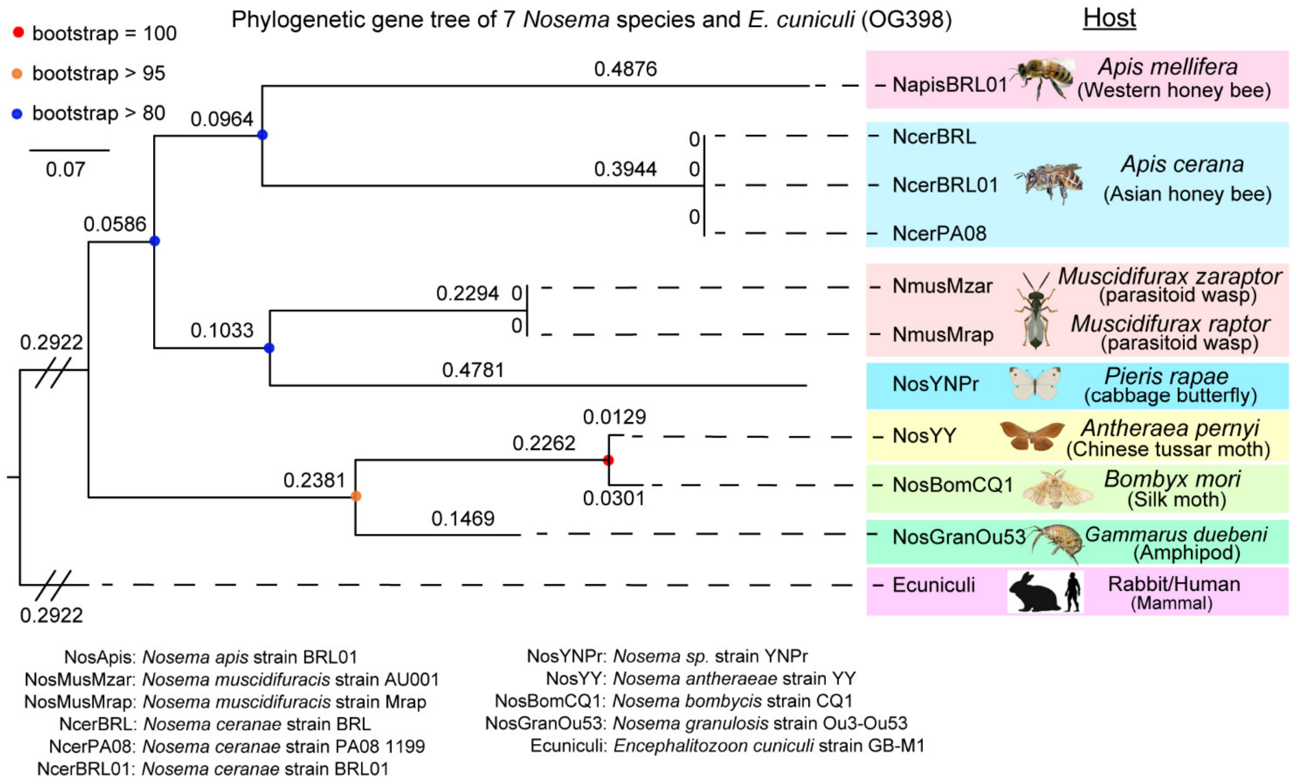

Figure S3. G-C content analysis of protein genes in *N. muscidifuracis*, *N. ceranae* and *E. cuniculi*.

The *x*-axis represents the proportion of G+C at the first two codon positions (GC12), and the *y*-axis represents the proportion of G+C at the third codon position (GC3).

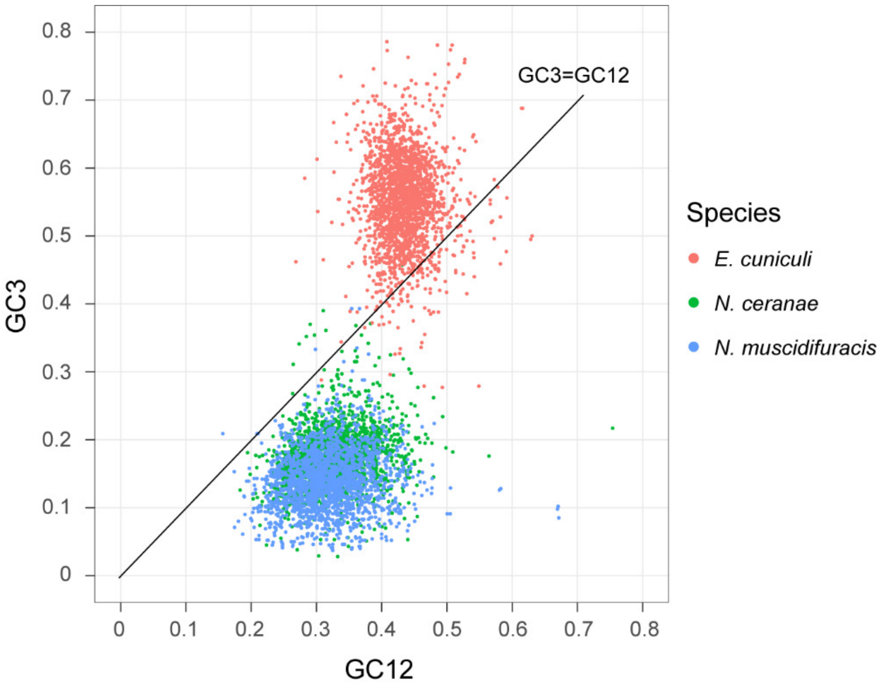

Figure S4. Codon usage frequencies of genes in genomes of seven *Nosema* species and *Encephalitozoon cuniculi*.

Each bar represents the proportion of codon usage in *Nosema* species and *E. cuniculi* protein coding genes, rank-ordered by the genome-wide average G-C content (from 24.8% to 47.5%). The codons were color-coded according to the number of G/C in them: 0, orange; 1, yellow; 2, blue; 3, green.

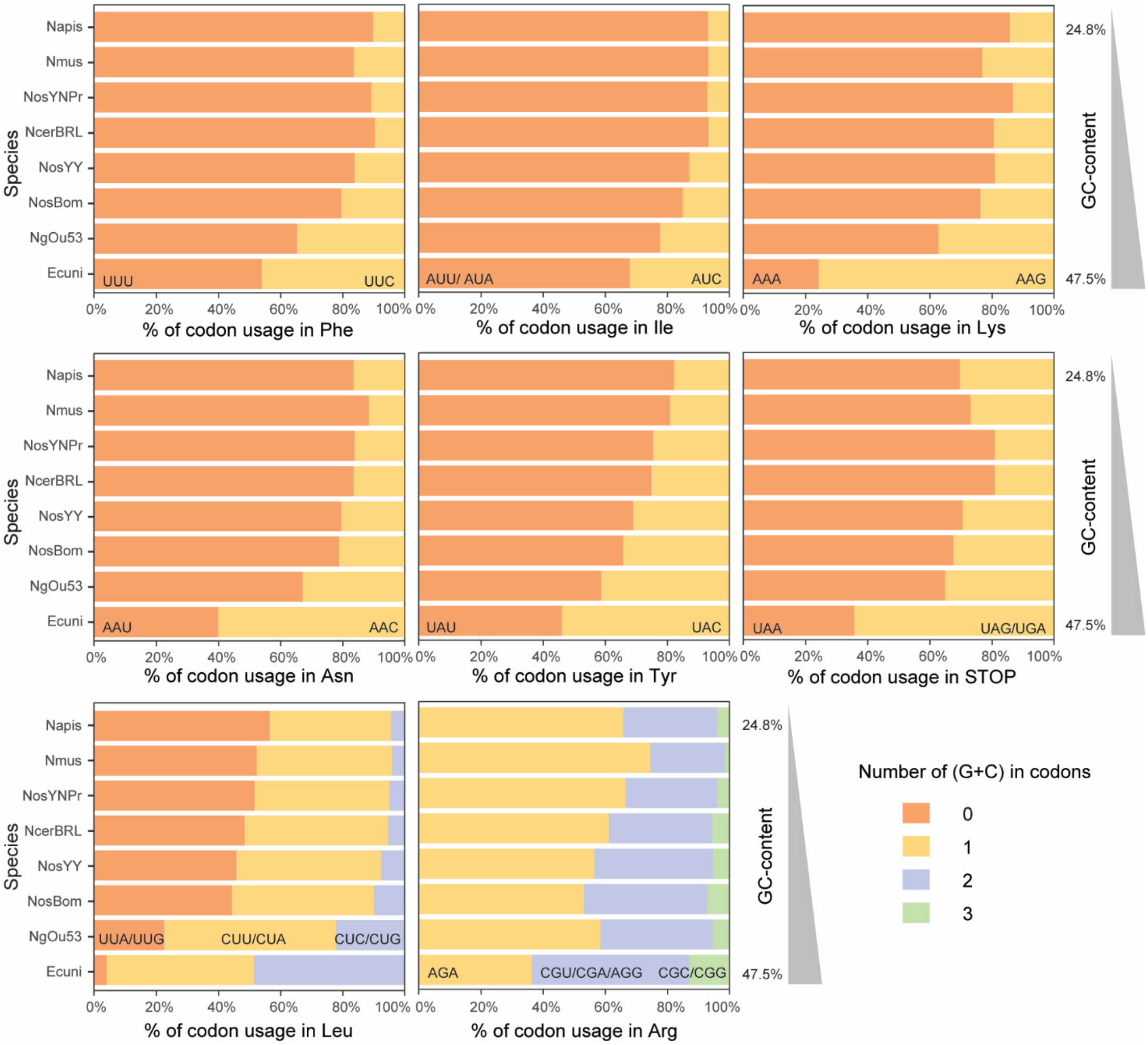

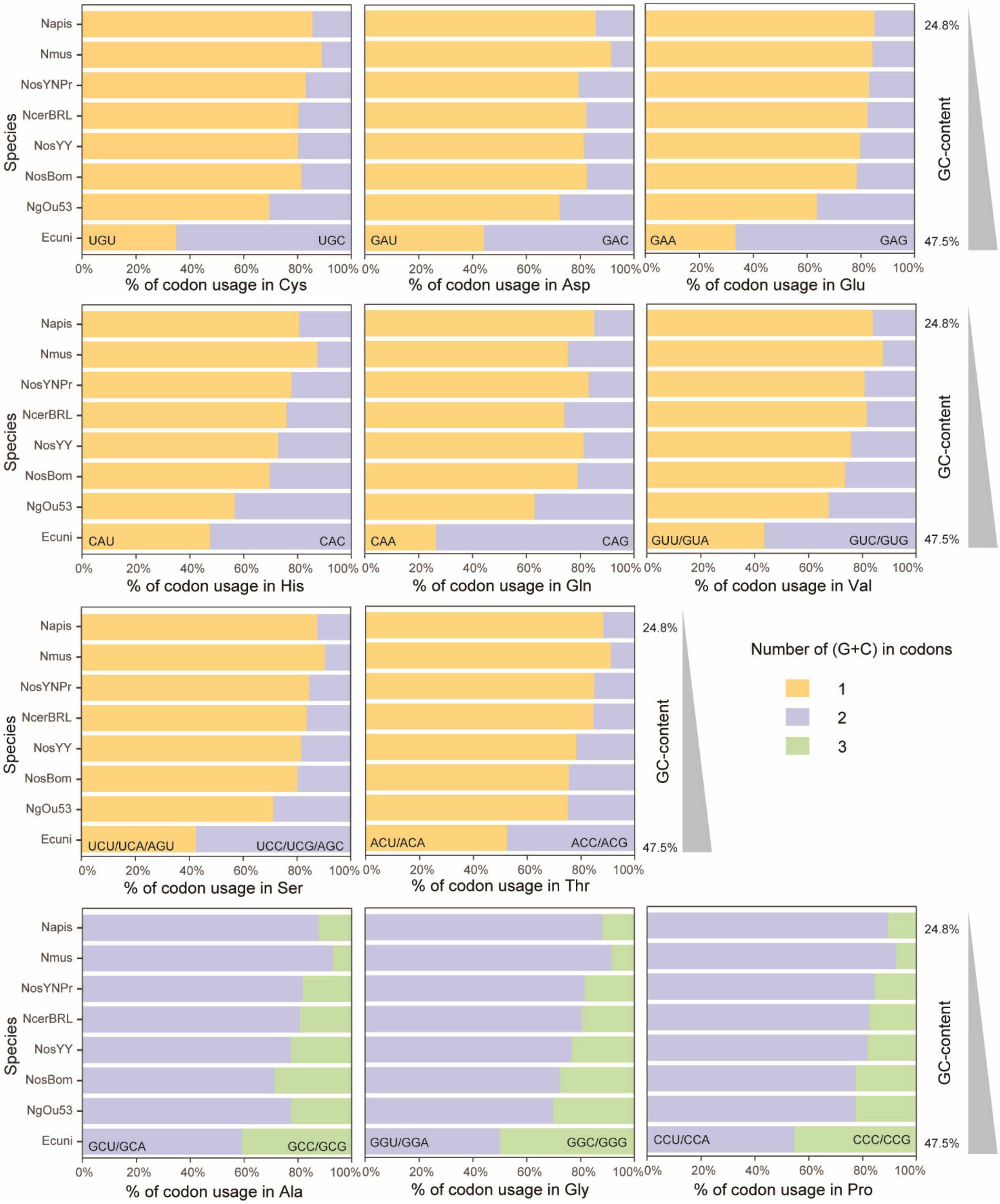
Species
Napis
Nmus
NosYNPr
NcerBRL
NosYY
NosBom
NgOu53
Ecuni
GC-content
24.8%
47.5%
0%
20%
40%
60%
80%
100%
UGU
UGC
% of codon usage in Cys
GAU
GAC
% of codon usage in Asp
GAA
GAG
% of codon usage in Glu
CAU
CAC
% of codon usage in His
CAA
CAG
% of codon usage in Gln
GUU/GUA
GUC/GUG
% of codon usage in Val
UCU/UCA/AGU
UCC/UCG/AGC
% of codon usage in Ser
ACU/ACA
ACC/ACG
% of codon usage in Thr
Number of (G+C) in codons
1
2
3
GCU/GCA
GCC/GCG
% of codon usage in Ala
GGU/GGA
GGC/GGG
% of codon usage in Gly
CCU/CCA
CCC/CCG
% of codon usage in Pro
