# Supplementary material for: Genome Structure, Evolution, and Host Shift of Nosema
Source: Biology (Basel). 2024 Nov 19;13(11):952. doi: 10.3390/biology13110952 (PMC11592040; doi:10.3390/biology13110952)
Supplement: Supplementary file 1 [file biology-13-00952-s001.zip › TableS1-S8_Nosema_genome_evolution_20240218.pdf]

Table S1. Accession numbers for *Nosema* genome assemblies analyzed in this study.

Species (strain)	Host name	Host order	Accession
N. muscidifuracis	*Muscidifurax zaraptor*	Hymenoptera	JAIOKI000000000
N. apis (BRL01)	*Apis mellifera*	Hymenoptera	GCA_000447185.1
N. ceranae (BRL)	*Apis cerana*	Hymenoptera	GCA_004919615.1
N. ceranae (PA08)	*Apis cerana*	Hymenoptera	GCA_000988165.1
N. ceranae (BRL01)	*Apis cerana*	Hymenoptera	GCA_000182985.1
Nosema sp. (YNPr)	*Pieris rapae*	Lepidoptera	NA*
N. antheraeae (YY)	*Antheraea pernyi*	Lepidoptera	NA*
N. bombycis (CQ1)	*Bombyx mori*	Lepidoptera	GCA_000383075.1
N. granulosis (Ou3-Ou53)	*Gammarus duebeni*	Amphipoda	GCA_015832245.1
E. cuniculi (GB-M1)	*Rabbit/Human*	Lagomorpha/ Primates	GCA_000091225.2

* , genome assembly and annotation obtained from personal communication with authors.

Table S2. Short-read genome sequencing data in *Nosema* species used for telomeric repeat motif identification.

Species (strains)	Data Accession numbers	Total number of reads	Candidate telomeric repeat motif	Sequencing platform
N. apis (BRL01)	SRX245851	493,431	N/A	454 GS FLX
N. ceranae (BRL)	SRX5338655	2,186,202	N/A	MinION
N. ceranae (PA08)	SRX318182	6,106,172	TTAGG	Illumina HiSeq 2000
N. ceranae (BRL01)	SRX003255	1,063,647	N/A	454 GS FLX
Nosema sp. (YNPr)	NA	Raw data not available		
N. antheraeae (YY)	NA	Raw data not available		
N. bombycis (CQ1)	SRX7209795	26,849,840	N/A*	Illumina HiSeq 2000
N. granulosis (Ou3-Ou53)	SRX5286701	7,327,280	N/A	Illumina MiSeq
E. cuniculi (GB-M1)	ERS610230	Raw data not available		

* cDNA was sequenced for this species (genomic data was not available).

Table S3. ***Encephalitozoon*** **telomeric repeat motifs inferred from long-read genome assemblies.**

Chromosome	*E. hellem* (GCA_029215505)	*E. intestinalis* (GCA_024399295)	*E. cuniculi* (GCA_027571585)
I	TTAGG	TTAGG	TTAGG
II	TTAGG	TTAGG	TTAGG
III	TTAGG	TTAGG	TTAGG
IV	TTAGG	TTAGG	TTAGG
V	TTAGG	TTAGG	TTAGG
VI	TTAGG	TTAGG	TTAGG
VII	TTAGG	TTAGG	TTAGG
VIII	TTAGG	TTAGG	TTAGG
IX	TTAGG	TTAGG	TTAGG
X	TTAGG	TTAGG	TTAGG
XI	TTAGG	TTAGG	Insufficient length

Table S4. Summary of the TRM call and composition of TAGG, TTAGG, and TAGGG repeat units in the telomere of *Nosema muscidifuracis*.

Scaffold ID	termini	TRM call	start	end	Length (bp)	TAGG (counts)	TTAGG (counts)	TAGGG (counts)
Nmus_SCAFFOLD01	end	TAGG/TTAGG	978765	982164	3400	734	91	2
Nmus_SCAFFOLD01	start	TAGG/TTAGG	1	1925	1925	378	73	10
Nmus_SCAFFOLD02	start	TAGG/TTAGG	1	3815	3815	799	114	10
Nmus_SCAFFOLD02	end	TAGG/TTAGG	929698	933025	3328	601	181	4
Nmus_SCAFFOLD03	end	TAGG/TTAGG	829614	833206	3593	504	147	8
Nmus_SCAFFOLD03	start	TAGG/TTAGG	1	2789	2789	735	102	29
Nmus_SCAFFOLD04	start	TAGG/TTAGG	1	3686	3686	717	160	4
Nmus_SCAFFOLD04	end	TAGG/TTAGG	678058	682066	4009	730	212	6
Nmus_SCAFFOLD06	end	TAGG/TTAGG	632849	635297	2449	539	106	4
Nmus_SCAFFOLD06	start	TAGG/TTAGG	1	2706	2706	505	84	2
Nmus_SCAFFOLD07	end	TAGG/TTAGG	609208	610585	1378	99	44	5
Nmus_SCAFFOLD07	start	TAGG/TTAGG	1	638	638	229	90	3
Nmus_SCAFFOLD08	end	TAGG/TTAGG	587055	588184	1130	134	45	1
Nmus_SCAFFOLD08	start	TAGG/TTAGG	1	763	763	245	30	0
Nmus_SCAFFOLD09	start	TAGG/TTAGG	1	2258	2258	431	107	0
Nmus_SCAFFOLD11	start	TAGG/TTAGG	1	2593	2593	510	92	19
Nmus_SCAFFOLD11	end	TAGG/TTAGG	542085	544348	2264	443	85	14
Nmus_SCAFFOLD12	start	TAGG/TTAGG	1	2006	2006	347	112	12
Nmus_SCAFFOLD13	start	TAGG/TTAGG	1	2395	2395	437	125	5
Nmus_SCAFFOLD14	start	TAGG/TTAGG	1	2064	2064	389	87	15
Nmus_SCAFFOLD14	end	TAGG/TTAGG	488377	490971	2595	459	142	10
Nmus_SCAFFOLD15	start	TAGG/TTAGG	1	1482	1482	275	76	1
Nmus_SCAFFOLD16	start	TAGG/TTAGG	1	751	751	148	31	1
Nmus_SCAFFOLD17	start	TAGG/TTAGG	1	2008	2008	349	111	12
Nmus_SCAFFOLD21	start	TAGG/TTAGG	1	1340	1340	263	56	2
Nmus_SCAFFOLD22	start	TAGG/TTAGG	1	836	836	142	53	1
Nmus_SCAFFOLD27	start	TAGG/TTAGG	1	3079	3079	545	175	5
Nmus_SCAFFOLD28	start	TAGG/TTAGG	1	1363	1363	264	61	1

Table S5. Number of genes in major functional pathways identified in *Nosema muscidifuracis* and *Saccharomyces cerevisiae*.

Pathway	*N. muscidifuracis* gene number	*S. cerevisiae* gene number
Oxidative phosphorylation	0	41
Endocytosis	11	50
mTOR signaling pathway	8	26
PI3K-Akt signaling pathway	7	19
Ribosome biogenesis in eukaryotes	23	56
Cellular senescence	8	19
Ubiquitin mediated proteolysis	17	39
Phagosome	6	13
Cell cycle	17	36
HIF-1 signaling pathway	6	12
Thyroid hormone signaling pathway	7	14
Protein export	8	16
Nucleotide excision repair	12	24
Proteasome	18	36
mRNA surveillance pathway	18	36
RNA degradation	15	30
Basal transcription factors	16	31
Mismatch repair	7	12
DNA replication	15	25
RNA polymerase	19	31
Tight junction	11	14
Wnt signaling pathway	8	10

Table S6. Motif sequences and significance identified in 449 shared orthologous genes in seven *Nosema* species and an outgroup species *Encephalitozoon cuniculi*.

Species (strain)	Predicted motif	E-value	Sites	Motif sequence logo (449 genes)
N. muscidifuracis	TTTTTTTACCCC	1.6e-256	342	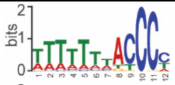
N. apis (BRL01)	ACCCT	7.4e-94	289	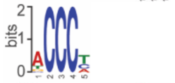
N. ceranae (BRL)	TTTTTTACCCCT	9.5e-174	227	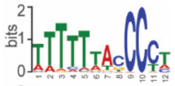
Nosema sp. (YNPr)	TTTTTTACCCC	2.6e-202	271	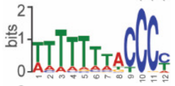
N. antheraeae (YY)	TTTTTTACCCCC	5.4e-155	226	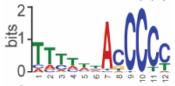
N. bombycis (CQ1)	TTTTTTACCCCC	1.6e-72	203	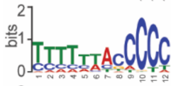
N. granulosis (Ou3-Ou53)	TTTTTTTACCCC	9.4e-172	273	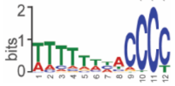
E. cuniculi (GB-M1)	TCTTTTTCTCCA	2.4e-18	449	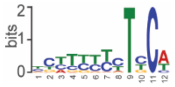

Table S7. Motif sequences and significance identified in predicted genes of seven *Nosema* species and *Encephalitozoon cuniculi*, excluding 449 shared orthologous genes.

Species (strain)	Predicted motif	E-value	Sites	Motif sequence logo (other genes)
N. muscidifuracis	TTTTTTTACCCC	4.8e-155	2263	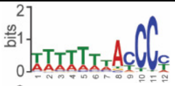
N. apis (BRL01)	ACCCT	2.7e+175	2220	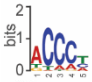
N. ceranae (BRL)	TTTTTTACCCCT	3.7e-21	1828	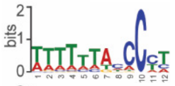
Nosema sp. (YNPr)	TTTTTTTACCCT	5.9e+217	1212	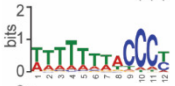
N. antheraeae (YY)	TTTACCCCC	8.0e+108	2354	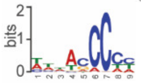
N. bombycis (CQ1)	CCCCA	5.9e+217	3692	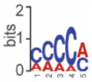
N. granulosis (Ou3-Ou53)	TTTTTAACCCC	2.4e+125	2819	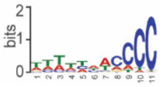
E. cuniculi (GB-M1)	TCTTCTTCTCCA	1.0e-6	1706	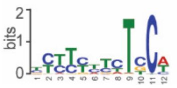

Table S8. Summary of codon usage in *Encephalitozoon cuniculi* and seven *Nosema* species.

Amino acid	Codon	*Ecuni*	*NgOu53*	*NosBom*	*NosYY*	*NosYNPr*	*Ncer*	*Nmus*	*Napis*
Asp	GAC	0.560305	0.291189	0.18309	0.202093	0.209223	0.180628	0.093042	0.150928
Asp	GAU	0.432633	0.706887	0.796095	0.790917	0.783527	0.817158	0.904751	0.846901
Cys	UGC	0.614226	0.278084	0.170369	0.188509	0.156869	0.184415	0.106656	0.136949
Cys	UGU	0.324098	0.659262	0.709667	0.730725	0.789482	0.757152	0.812064	0.789245
Glu	GAG	0.648483	0.355568	0.217161	0.205787	0.169707	0.178764	0.167554	0.148869
Glu	GAA	0.349163	0.641958	0.765158	0.787482	0.824976	0.819466	0.832446	0.847513
His	CAC	0.497706	0.39379	0.276413	0.258217	0.200857	0.231637	0.129744	0.173927
His	CAU	0.4538	0.553998	0.62108	0.677067	0.730994	0.702847	0.786034	0.750096
Lys	AAG	0.743067	0.369549	0.242592	0.224235	0.159673	0.196338	0.234493	0.145501
Lys	AAA	0.253637	0.628253	0.754274	0.773435	0.838877	0.803662	0.764771	0.854499
Phe	UUC	0.464554	0.335312	0.208835	0.165487	0.111067	0.099906	0.180293	0.10354
Phe	UUU	0.521792	0.657818	0.781989	0.829076	0.887	0.895668	0.816397	0.884883
Asn	AAC	0.589207	0.343451	0.226821	0.21782	0.165795	0.174984	0.132891	0.167739
Asn	AAU	0.396669	0.65545	0.768031	0.77778	0.832755	0.823688	0.867109	0.831538
Gln	CAG	0.714801	0.360494	0.201294	0.195022	0.163611	0.258393	0.25422	0.143114
Gln	CAA	0.260247	0.622193	0.751929	0.775985	0.817539	0.718145	0.706059	0.829751
Ile	AUC	0.327146	0.221262	0.154651	0.131936	0.072403	0.069735	0.072335	0.070137
Ile	AUA	0.365913	0.36909	0.304799	0.316017	0.37453	0.402943	0.464562	0.415214
Ile	AUU	0.305529	0.407999	0.539208	0.548682	0.552101	0.526879	0.462	0.513563
Ter*	UAG	0.27354	0.165155	0.104969	0.112348	0.090382	0.10093	0.130563	0.130608
Ter*	UGA	0.369586	0.156636	0.192704	0.181724	0.100048	0.088092	0.136815	0.166064
Ter*	UAA	0.356874	0.598791	0.62444	0.704634	0.80522	0.810978	0.732622	0.683068
Tyr	UAC	0.521315	0.402925	0.343767	0.318685	0.243406	0.254941	0.207976	0.177533
Tyr	UAU	0.460794	0.590205	0.635642	0.668113	0.750794	0.739304	0.783197	0.814869
Ala	GCC	0.253953	0.156899	0.178943	0.143513	0.093054	0.092261	0.044511	0.057132
Ala	GCG	0.148465	0.066785	0.078384	0.078418	0.073726	0.087301	0.022415	0.049773
Ala	GCA	0.409193	0.364352	0.289777	0.329127	0.363117	0.421871	0.330499	0.407013
Ala	GCU	0.185093	0.404544	0.397838	0.417619	0.428537	0.370678	0.538581	0.410105
Gly	GGG	0.299298	0.172667	0.160874	0.118914	0.118488	0.098811	0.073795	0.064093
Gly	GGC	0.189367	0.125047	0.11796	0.121421	0.065931	0.099047	0.021409	0.050368
Gly	GGU	0.095133	0.266675	0.310359	0.328508	0.340053	0.333239	0.326952	0.38225
Gly	GGA	0.412906	0.42874	0.382831	0.412518	0.454261	0.451639	0.554674	0.466024
Pro	CCG	0.222561	0.077764	0.097127	0.090231	0.061248	0.077862	0.025737	0.050995
Pro	CCC	0.222154	0.153555	0.116009	0.087111	0.08323	0.084267	0.049185	0.043542
Pro	CCA	0.27272	0.42923	0.335977	0.358798	0.384907	0.384039	0.422367	0.440829
Pro	CCU	0.261379	0.316093	0.404333	0.423994	0.428565	0.417089	0.451957	0.399873
Thr	ACC	0.234751	0.150608	0.128496	0.106241	0.071719	0.070121	0.04868	0.055339
Thr	ACG	0.231929	0.100129	0.123345	0.112713	0.070596	0.077802	0.039502	0.056813
Thr	ACU	0.17608	0.315996	0.381003	0.379569	0.398137	0.362124	0.46772	0.376398
Thr	ACA	0.348295	0.431069	0.358427	0.393711	0.454231	0.488182	0.436742	0.506385

*Ter: stop codon.

**Amino acid**	**Codon**	***Ecuni***	***NgOu53***	***NosBom***	***NosYY***	***NosYNPr***	***Ncer***	***Nmus***	***Napis***
Val	GUG	0.335319	0.182066	0.134306	0.126298	0.091794	0.114373	0.065342	0.09938
Val	GUC	0.231866	0.136449	0.128617	0.11597	0.094845	0.068727	0.064313	0.057523
Val	GUA	0.15122	0.359824	0.32018	0.321433	0.384852	0.408526	0.472327	0.363291
Val	GUU	0.277358	0.320287	0.410407	0.43164	0.425126	0.403062	0.389559	0.474379
Arg	CGG	0.077168	0.026784	0.031148	0.02479	0.018698	0.023738	0.008721	0.017023
Arg	CGC	0.046414	0.025959	0.03545	0.026308	0.01827	0.030412	0.004394	0.015549
Arg	CGA	0.045391	0.087771	0.096186	0.104102	0.084821	0.095435	0.059561	0.094674
Arg	CGU	0.033967	0.074319	0.098086	0.103598	0.082625	0.092565	0.085328	0.098571
Arg	AGG	0.423228	0.198371	0.194247	0.176544	0.124201	0.140705	0.096281	0.098345
Arg	AGA	0.371949	0.58185	0.522278	0.552491	0.657368	0.60475	0.728429	0.654131
Leu	CUG	0.313443	0.105483	0.047279	0.035159	0.025581	0.035123	0.018216	0.020672
Leu	CUC	0.176279	0.102944	0.058497	0.045208	0.026438	0.020828	0.028375	0.022394
Leu	CUA	0.103045	0.158118	0.074823	0.081661	0.098789	0.11123	0.087605	0.08164
Leu	CUU	0.252172	0.204517	0.221797	0.223341	0.184952	0.216386	0.246413	0.163481
Leu	UUG	0.113738	0.191194	0.162048	0.17068	0.159104	0.139748	0.119266	0.152873
Leu	UUA	0.040852	0.23692	0.434214	0.44188	0.505136	0.4758	0.499021	0.558217
Ser	AGC	0.255461	0.123442	0.059184	0.067141	0.054004	0.07124	0.040435	0.054871
Ser	UCC	0.169016	0.094551	0.079375	0.0587	0.050944	0.036587	0.029131	0.031743
Ser	UCG	0.155796	0.072731	0.068268	0.068997	0.047859	0.050937	0.027334	0.039648
Ser	UCU	0.195107	0.285731	0.317945	0.325763	0.316944	0.320324	0.330282	0.282975
Ser	UCA	0.100219	0.21325	0.216928	0.232229	0.242136	0.233638	0.276536	0.281372
Ser	AGU	0.124401	0.209746	0.253823	0.243546	0.287146	0.284175	0.291501	0.306135
Met*	AUG	1	1	1	1	1	1	1	1
Trp	UGG	1	1	1	1	1	1	1	1

*Met: start codon.
